# Sex differences in rat placental development: from pre-implantation to late gestation

**DOI:** 10.1186/s13293-017-0138-6

**Published:** 2017-05-16

**Authors:** J. I. Kalisch-Smith, D. G. Simmons, M. Pantaleon, K. M. Moritz

**Affiliations:** 1 School of Biomedical Sciences, The University of Queensland, St Lucia, QLD 4072 Australia; 2 Centre for Child Health Research, The University of Queensland, South Brisbane, QLD 4101 Australia

**Keywords:** Blastocyst, Trophectoderm, Placenta, Trophoblast, Sexual dimorphism, Differentiation, DOHaD

## Abstract

**Background:**

A male fetus is suggested to be more susceptible to *in utero* and birth complications. This may be due in part to altered morphology or function of the XY placenta. We hypothesised that sexual dimorphism begins at the blastocyst stage with sex differences in the progenitor trophectoderm (TE) and its derived trophoblast lineages, as these cells populate the majority of cell types within the placenta. We investigated sex-specific differences in cell allocation in the pre-implantation embryo and further characterised growth and gene expression of the placental compartments from the early stages of the definitive placenta through to late gestation.

**Methods:**

Naturally mated Sprague Dawley dams were used to collect blastocysts at embryonic day (E) 5 to characterise cell allocation; total, TE, and inner cell mass (ICM), and differentiation to downstream trophoblast cell types. Placental tissues were collected at E13, E15, and E20 to characterise volumes of placental compartments, and sex-specific gene expression profiles.

**Results:**

Pre-implantation embryos showed no sex differences in cell allocation (total, TE and ICM) or early trophoblast differentiation, assessed by outgrowth area, number and ploidy of trophoblasts and P-TGCs, and expression of markers of trophoblast stem cell state or differentiation. Whilst no changes in placental structures were found in the immature E13 placenta, the definitive E15 placenta from female fetuses had reduced labyrinthine volume, fetal and maternal blood space volume, as well as fetal blood space surface area, when compared to placentas from males. No differences between the sexes in labyrinth trophoblast volume or interhaemal membrane thickness were found. By E20 these sex-specific placental differences were no longer present, but female fetuses weighed less than their male counterparts. Coupled with expression profiles from E13 and E15 placental samples may suggest a developmental delay in placental differentiation.

**Conclusions:**

Although there were no overt differences in blastocyst cell number or early placental development, reduced growth of the female labyrinth in mid gestation is likely to contribute to lower fetal weight in females at E20. These data suggest sex differences in fetal growth trajectories are due at least in part, to differences in placenta growth.

**Electronic supplementary material:**

The online version of this article (doi:10.1186/s13293-017-0138-6) contains supplementary material, which is available to authorized users.

## Background

Exposure of the fetus to a sub-optimal in utero environment such as poor maternal nutrition, stress or hypoxia can alter its development, potentially in a sexually dimorphic manner [1]. Male fetuses are often reported to be at greater risk of preterm birth, neonatal complications and prenatal mortality [2, 3]. For this reason, it is commonly viewed that males are more susceptible to developmental perturbations than females [4]. Critical to normal growth and viability of the fetus is adequate development of the mature chorioallantoic placenta. Males often exhibit larger placental growth (weight) than females in uncomplicated human pregnancies at term [5]. This is thought to help facilitate the greater growth trajectory of the male fetus. Sexual dimorphism in placental development is most often examined following prenatal stressors [6, 7], but inherent differences have rarely been investigated. Study of the placenta is normally restricted to analysis at late gestation or term. Interestingly, sex-specific changes have been observed in the late gestation placenta of the rat in a model of alcohol exposure which only occurred during the peri-conception, prior to implantation [8]. The question then arises to when does sexual dimorphism in growth of the placenta emerge, and can it be traced back to the pre-implantation embryo?

The placenta and its associated trophoblast subtypes derive from the trophectoderm (TE) of the pre-implantation embryo. In the rodent, a subset of cells from the TE differentiate into invasive trophoblast giant cells, which migrate into the maternal uterine compartment, while the remaining TE cells form later in gestation to populate either the labyrinth or junctional zone compartments [9, 10]. The labyrinth contains fetal and maternal vasculature for the two-way exchange of nutrients and waste products, while the junctional zone is thought to have a primary endocrine role [11]. Placentas from males and females can alter their growth in a sex-specific and zone-specific manner when confronted with maternal perturbations in different critical windows, which can lead to sex-specific alterations in fetal viability (recently reviewed by us in [1]).

Investigations of sex differences in the early embryo have been largely restricted to analysis of in vitro models which use superovulation to generate large numbers of embryos. Both hormone-induced ovulation and in vitro culture techniques are implicated in causing stress to the early embryo, influencing its development [12, 13] and therefore may be potential confounders for the determination of basal sex differences. Current analysis in vitro suggests male mouse [14–18] and bovine [19–21] embryos develop to the blastocyst stage at a faster rate than female embryos. Quantification of cell number in vitro supports this outcome, with males displaying increased total, TE or ICM count [21–23]. However, there is conflicting evidence in human [23–25], bovine [26–28], porcine [29, 30], in vitro [31] and in vivo derived mouse models [17], which rather show equal developmental rates between the sexes. Indeed, culture in vitro has been shown to increase cell death in the early embryo, and therefore, may suggest that the reported sex differences are not in embryonic growth, but in differential survival from an artefact of culture. Sexual dimorphism is also suggested to exist in blastocyst physiology. Metabolic rate [32] and stress responses [18] are suggested to be heightened in males, while females seem to be more susceptible to cell death [21, 22]. Expression profiles of bovine male and female blastocysts show sexual dimorphism of both X-linked and autosomal genes [33, 34], whilst sexual dimorphism has also been shown in expression profiles as early as the 8-cell stage in the mouse [35].

In this study, we use naturally cycling rat dams (without superovulation) to characterise sex differences in the pre-implantation embryo, through to trophoblast differentiation, and the formation of the placental architecture. This allowed us to examine inherent sex differences without prenatal stressors and highlights the importance of appropriately derived animals to investigate these outcomes. We hypothesised inherent sex differences exist in the blastocyst stage with differences in the progenitor trophectoderm (TE) and its derived trophoblast lineages which result in sex differences in growth of the definitive placenta.

## Methods

### Ethics

All animal experiments and procedures were approved by The University of Queensland Animal Ethics Committee (AEC approval number SBS/022/12/NHMRC) prior to commencement of this study.

### Animal treatment, embryo collection and culture

Dam weight was at least 230 g before entering the protocol. Sprague Dawley dams were maintained on standard laboratory chow. In addition, tissues (blastocysts and placentas) were available from dams fed either chow or a control liquid diet used previously as a control for ethanol fed dams [8, 36]. Dams were time-mated over a 5-h window from 1200 to 1700 h. The following day was considered E1. At 0900 h on E5, a subset of dams (*N* = 17) were rapidly killed via guillotine prior to dissection of the uterine horns. Oviducts and uteri were flushed in Hepes-KSOM media [37] and embryos allocated to either staining or culture procedures.

### Staining procedures for pre-implantation embryos

Embryos were prepared for immunohistochemistry as previously described [38]. Briefly, embryos were fixed in 2% paraformaldehyde in 1× PBS for 20 min at room temperature (RT), were washed in PBS and were immobilized on Cell-Tak (Collaborative Biomedical Products)-coated coverslips. Embryos were permeabilised in 0.25% Triton in PBS and were washed before blocking with 10% normal goat serum/BSA/Tween-20/PBS (PBT) for 1 h RT. Embryos were incubated in 1:50 anti-Mouse CDX2 (BiogenX, MU392A-UC) overnight at 4 °C. Following, embryos were extensively washed in PBT prior to exposure to goat anti-mouse secondary antibody labelled with Alexa 488 (1:1000, Molecular Probes) for 1 h RT. Embryos were counterstained with DAPI (Sigma Aldrich Inc, B2388) in PBS for 15 min and were transferred through increasing concentrations of glycerol in PBS before mounting with Vectashield (Vector Labs, H-1000).

### Blastocyst outgrowth assays

E5 embryos were placed into pre-heated microdrops of trophoblast stem cell (TS) media on tissue culture plates or gelatine-coated coverslips and cultured in a humidifying chamber (Cook, Australia) at 37 °C 5% CO_2_/5% O_2_/90% N_2_ under paraffin oil. They were cultured for 6 days, with media changed every 2 days, and imaged with an inverted phase contrast microscope (Leica). Areas occupied by outgrowths were traced in ImageJ using calibrated settings. A subset of outgrowths was immunolabelled as described for staining procedures above with anti-rabbit pan-cytokeratin (DAKO, Z0622) to distinguish cytoskeleton, goat anti-rabbit Alexa 568 secondary antibody and DAPI (Sigma Aldrich Inc, B2388) to assess number and ploidy of trophoblast nuclei. The remaining outgrowths were collected and extracted for DNA and RNA with Trizol (Life Technologies) and cDNA synthesis from Quantitek reverse transcription kit (Qiagen) using as per manufacturer’s protocol.

### Confocal microscopy, image analysis and quantification

Embryos and outgrowths were visualised using an inverted microscope (Leica, DMi8). Pre-implantation embryos utilised a 40× air objective and captured in Z stacks of 40, while outgrowths were imaged at 20× in Z stacks of 10 using a tile scan. Excitation filters of 488 nm (green), 561 nm (red) and 405 nm (blue) fluorescence were imaged separately. CDX2 immunoreactivity (green) was localised to the TE of the E5 blastocyst. Cells devoid of CDX2 immunoreactivity were assumed to be ICM. TE and ICM cells were counted individually using ImageJ (NIH). ICM proportion was calculated as ICM (%) = (number of ICM cells/number of total cells) × 100.

For trophoblast outgrowths, Z stacks of the DAPI channel were compressed using the average intensity for manual cell counts and ploidy analysis using ImageJ. Smallest trophoblasts with positive staining for pan-cytokeratin had nuclear sizes exceeding 200 um^2^. ICM cells (2N) were found to be less than 200 um^2^ in size. Nuclear sizes above 1000 um^2^ were considered P-TGCs. DNA content, a surrogate marker of ploidy, was quantified from fluorescent intensity of nuclear DAPI. DNA content of all trophoblasts and P-TGCs were normalised to ICM cells of the same image to obtain relative ploidy to 2N.

### Genotyping for sex of pre-implantation embryos and blastocyst outgrowths

After analysis, the pre-implantation embryos and blastocyst outgrowths were removed mechanically from coverslips and were digested overnight at 55 °C in 5 ul of lysis buffer (50 mM KCl, 10 mM Tris-HCl pH 8.3, 2.5 mM MgCl_2_.6H_2_O, 0.1 mg/ml gelatin, 0.45% v/v Nonidet P40, 0.45% v/v Tween 20) with 1 ul of 20 mg/mL proteinase K. Proteinase K was then inactivated for 10 min at 95 °C. The total 6 ul sample was used to amplify male determining gene *Sry* and *B*-*Actin* as a control. Amplification of both genes was carried out in the same reaction due to limited DNA content. Primers for Sry (317 bp) and B-Actin (220 bp) were derived from Miyajima (2009), ref [39] for use in the rat (see Additional file 1 for primer sequences and PCR conditions). A 2% agarose gel was run for 35 min at 100 V to separate the bands. Double bands indicated male embryos, and single bands represented female embryos. A number of samples (~10%) failed to result in clear bands and were eliminated from analysis.

### Collection of placental tissues at mid- and late-gestation

A subset of dams was maintained for collection of fetal and placental tissues at E13 (4 litters), E15 (9 litters) and E20 (14 litters). For E13, dams were sacrificed by guillotine as above. E15 and E20 dams were heavily anaesthetised with 50:50 ketamine: xylazil as previously described [8]. Fetal and placental weights were taken at post-mortem for E15 and E20 cohorts, and placentas were separated from the uterus, with the junctional zone and labyrinth and weighed separately. In addition, a ratio of placental weight to body weight was calculated to estimate placental efficiency. A subset of placentas was snap frozen in liquid nitrogen prior to RNA extraction. Other placental samples were cut in half with uterus and decidua attached and were fixed in 4% paraformaldehyde prior to processing into paraffin for stereology. It was assumed that tissue shrinkage was even between groups. A subset of labyrinth samples from E15 to E20 placentas were cut into 1 mm^3^ and were fixed into 2.5% glutaraldehyde in 0.2 M sodium cacodylate buffer until tissue processing for transmission electron microscopy. Fetal tissue for genotyping for sex was collected as previously described [8].

### Stereology for placental volumes

Placental halves were sectioned exhaustively at 5 um for the collection of 5 equally spaced sections. Unbiased stereology for placental volumes and surface areas was carried out as described by [11]. To clearly localise fetal blood spaces within the labyrinth at each individual age, different histological procedures were used. At E13, nucleated fetal blood cells were easy to distinguish with haematoxylin and eosin staining, while this was not appropriate at either E15 or E20. At E15, the in situ hybridisation marker *Mest* clearly identified the fetal endothelial cells (see Additional file 1 for primer sequences) [40], while at E20, the endothelial marker Isolectin B4 (Sigma Aldrich, L5391) was used as per manufacturer’s instructions. Neither of these markers were appropriate at other ages. By doing so, all compartments of the placenta (decidua, junctional zone and labyrinth) and the fetal (FBS) and maternal blood spaces (MBS) were quantified. One or two placentas of each sex from each litter were used for analysis. For volume analyses, we utilised 7 males and 6 females at E13 (4 litters), 9 males and 8 females from 6 litters at E15 and 12 males and 11 females from 6 litters at E20.

### Transmission electron microscopy

Labyrinth samples from E15 and E20 placentas were cut into 1 mm^3^ were collected and fixed into 2.5% glutaraldehyde in 0.2 M sodium cacodylate buffer until tissue processing. All samples were washed in 0.1 M sodium cacodylate buffer at 250 W and were fixed further in 1% osmium tetroxide in cacodylate buffer at 80 W under vacuum in a Biowave (Pelco). Samples were rinsed with water at 250 W prior to counterstaining in 2% uranyl acetate _(aq)_ at 150 W under vacuum to enhance contrast. Samples were dehydrated in ethanol series (30, 50, 70, 90, 100, and 100%) at 250 W and were infiltrated in increasing series of epon resin (1:3, 1:1, 3:1, 100%, 100%) under vacuum. Resin was polymerised for 48 h at 60 °C. Thick sections were cut at 500 nm and were stained with Toluidine blue to confirm localisation in the labyrinth. Thin sections of 100 nm were post-stained with lead citrate prior to visualisation with an electron microscope (Jeol JEM-101). Two blocks per labyrinth samples were used to gather a minimum of six interhemal membranes (IHM) of which were averaged per block, with a total average for each animal.

### Gene expression studies

Total RNA from labyrinth samples at E13 and E15 were extracted using RNeasy kits with on column DNase digestion (Qiagen). 1 ug RNA was reverse transcribed using the iScript cDNA kit (Bio-Rad). qRT-PCR was performed using TaqMan (Qiagen) or SYBR green (Qiagen) reagents, with 100 ng cDNA used per reaction. Expression profiles of two replicates per sample were analysed relative to the geometric mean of *18S*, *Rplp0* and *Rpl13a* for outgrowths, while E13 whole placenta and E15 labyrinth cDNA used *18S* and *Rpl13a*. Relative gene expression was determined in outgrowths for genes associated with trophoblast stem cell maintenance (*Eomes*) and early TGC differentiation (*Ascl2*, *Prl3d1* and *Hand1*). Placental labyrinth samples were examined for mRNA expression of trophoblast differentiation markers (*Mest*, *Syna* and *Gcm1*), and genes associated with placental growth and vasculogenesis (*Igf1*, *Igf2*, *Vegfa* and *Pgf*), their respective receptors (*Igf1r*, *Igf2r*, *Kdr* and *s*-*Flt*). See Additional file 1 for primer information.

### Statistical analysis

For embryo studies, statistically significant differences (*P < 0.05; **P < 0.01) were analysed using Student’s *t* tests to examine sex differences. All pre-implantation embryos were pooled for analyses. To analyse changing outgrowth area between sexes over time, repeated measures ANOVA was used with litter averages used as each litter was cultured separately. Subsequent outgrowth analyses also used litter averages for cell counts, ploidy and gene expression assays. For analysis of placental morphology, a two-way ANOVA with sex and zone/compartment as factors was used, with Tukey’s post-hoc analysis used where appropriate. As expected, this revealed a strong effect of zone and thus subsequent analysis testing for sex differences was performed at each individual age using a Student’s *t* test. When Student’s *t* tests showed unequal variances, Mann-Whitney’s non-parametric tests were used. Mean relative gene expression of treatments were standardised to the control male group, displayed as mean ± standard error of the mean. Gene expression studies from E13 and E15 were analysed by using a two-way ANOVA where ‘gene’ and ‘sex’ were factors. All graphs and statistics were performed using GraphPad Prism 6 software (GraphPad Software, Inc., San Diego, CA, USA).

## Results

### Embryo studies

**Fig. 1:**
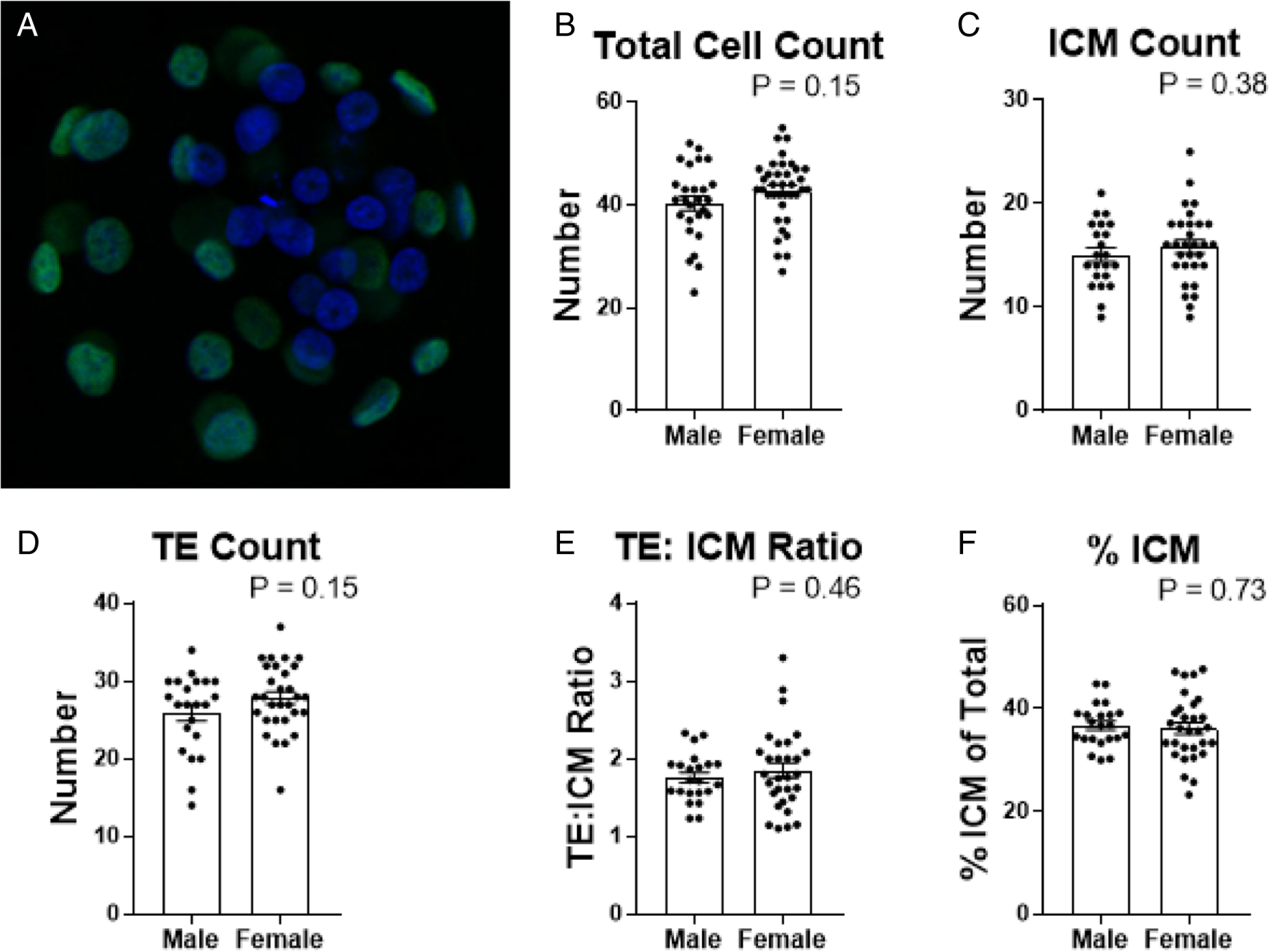
Analysis of pre-implantation development at E5 for male and female in vivo-derived embryos. **a** Representative image of E5 blastocyst immunolocalised with CDX2 (*green*) marking the trophectoderm, and the counterstain for nuclei with DAPI (*blue*). **b** Total cell count. **c** Inner cell mass *(ICM)* count. **d** Trophectoderm (*TE*) count. **e** Ratio of trophectoderm to inner cell mass (*TE:ICM*). **f** Percentage of inner cell mass to total cell count (*% ICM*). Data shows mean ± SEM, analysed by Student’s *t* test. Pooled embryos from *N* = 22–27 male, *N* = 30–37 female from 11–13 litters

**Fig. 2:**
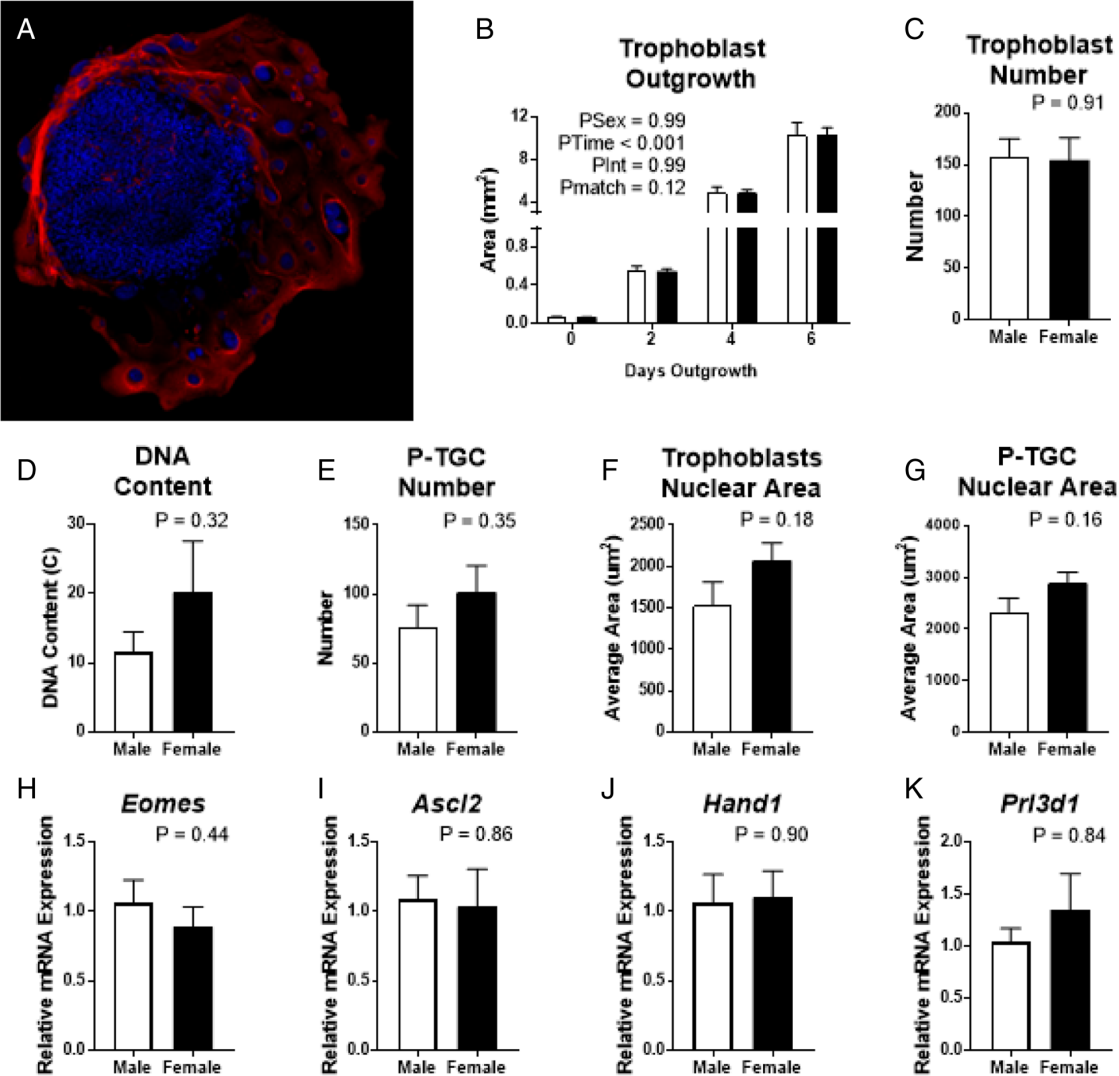
Gene profiles of trophoblast outgrowths after 6 days of culture from in vivo-derived dams. All data are presented as mean ± SEM, analysed by Student’s *t* test. *White* (male), *black* (female). **a** Representative image of day 6 cultured trophoblast outgrowth from E5 stained with trophoblast-specific marker pan-cytokeratin (*red*) and DAPI (*blue*). **b** Trophoblast outgrowth area over time from time 0 (E5-derived embryo). Assessed at day 6 of culture were **c** number of trophoblasts (nuclear area > 200um^2^), **d** DNA content relative to 2N ICM cells, **e** parietal trophoblast giant cell (P-TGC) number (nuclear area > 1000 um^2^), **f** trophoblast nuclear area, **g** P-TGC nuclear area and **h**–**k** expression profiles. **h-k** gene expression profiles displayed show the gene of interest relative to the geometric mean of 3 housekeeper genes (*Rplp0*, *Rpl13a*, *18S*). Expression of *Prl3d1* **k** showed unequal variance and was analysed by Mann-Whitney non-parametric test. Data in **b** show litter averages from *N* = 4 litters, 2–9/sex/litter. Cell counts and DNA content (**c**–**g**) show litter averages of *N* = 4 litters, 1–3/sex/litter. QPCR (**h**–**k**) show *N* = 8/sex from 4 litters

To localise the distinct cell lineages of the pre-implantation embryo, CDX2 immunoreactivity was used to positively label the trophectoderm (TE), with all cell nuclei marked with DAPI as shown in Fig. 1a. Cells negative for CDX2 staining were counted as ICM cells. No differences were observed between embryos from dams on a chow diet compared to those on a liquid diet. When male and female embryos were compared, no differences were found in total, ICM or TE cell numbers (Fig. 1b–d), or when expressed as percentages or ratios (Fig. 1e, f). When E5 embryos from naturally cycling dams were cultured for 6 days, trophoblasts outgrowths showed no differences between sexes for outgrowth area, number of trophoblasts, P-TGCs, their nuclear area or DNA content (Fig. 2a–g). This result was also reflected in outgrowth gene expression profiles for markers of trophoblast stem cell maintenance and differentiation (Fig. 2h–k).

### Placental biometry at E15 and E20

E15 and E20 fetal and placental weights and dimensions are shown in Table 1. Although all parameters increased in weight with age (PAge < 0.0001 for all parameters), there were no sex differences in placental length, width, or depth were found, nor weights of labyrinth or junctional zone compartments. Overall, placental weight at E15 and E20 was lower in females (PSex = 0.05) being 12% lower at E15 and 5% lower at E20. Fetal body weight also differed between the sexes (PSex < 0.05), but there was also an interaction with age (PInt < 0.05). Post hoc analysis demonstrated no significant difference in fetal body weight at E15, but males were heavier than females by E20. No changes to placental efficiency (placental weight:body weight ratio) were exhibited at either age.

**Table 1:** Fetal weight and placental weight and dimensions in males and females at E15 and E20

Variables	Male E15	Female E15	Male E20	Female E20	*Statistics*
Fetal weight (g)	0.208 ± 0.007	0.2062 ± 0.007	2.676 ± 0.038^†^	2.534 ± 0.035^†^	PSex < 0.05
					PAge < 0.0001
					PInt < 0.05
Placental weight (g)	0.247 ± 0.023	0.217 ± 0.006	0.561 ± 0.01221	0.536 ± 0.012	PSex = 0.05
					PAge < 0.0001
					PInt = 0.86
Placenta:BW ratio (g/gbw)	1.128 ± 0.059	1.127 ± 0.041	0.181 ± 0.010	0.188 ± 0.011	PSex = 0.92
					PAge < 0.0001
					PInt = 0.89
Labyrinthine wet weight (g)	0.0863 ± 0.009	0.094 ± 0.006	0.317 ± 0.018	0.306 ± 0.018	PSex = 0.93
					PAge < 0.0001
					PInt = 0.57
Junctional zone wet weight (g)	0.102 ± 0.008	0.096 ± 0.006	0.184 ± 0.019	0.196 ± 0.022	PSex = 0.88
					PAge < 0.0001
					PInt = 0.62
Placental length (mm)	12.090 ± 0.352	12.140 ± 0.232	14.080 ± 0.267	13.910 ± 0.196	PSex = 0.84
					PAge < 0.0001
					PInt = 0.69
Placental width (mm)	11.410 ± 0.245	11.040 ± 0.187	12.650 ± 0.216	12.590 ± 0.241	PSex = 0.38
					PAge < 0.0001
					PInt = 0.52
Placental depth (mm)	2.979 ± 0.104	2.833 ± 0.079	4.187 ± 0.086	4.056 ± 0.092	PSex = 0.16
					PAge < 0.0001
					PInt = 0.94

All data are presented as mean ± SEM and analysed by two-way ANOVA for sex with age. †shows significant difference *P* < 0.01 between sexes from Tukey’s post-hoc analysis. E15 parameters – *N* = 9 litters, E20 parameters – *N* = 12-14 litters

### Placental morphology from early to late gestation

**Fig. 3:**
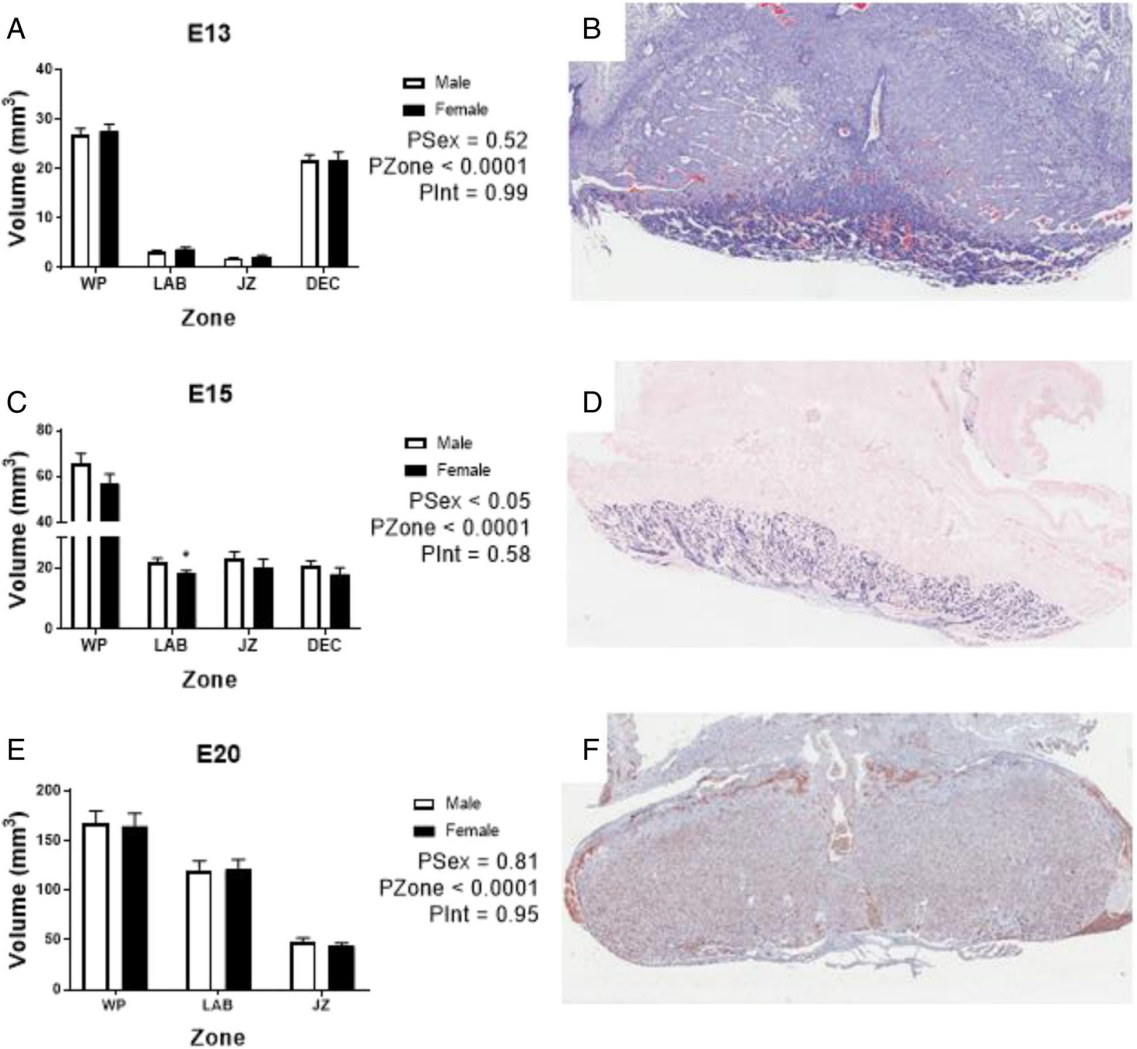
Sexually dimorphic outcomes of zonal volumes within the placenta from E13 to E20 during gestation. Volumes of placental zones. *WP* whole placenta, *LAB* labyrinth, *JZ* junctional zone, *DEC* decidua were estimated at E13 (**a**), E15 (**c**) and E20 (**e**). **b** Haematoxylin and eosin stain of an E13 placenta. **d** In situ hybridisation for *Mest* at E15 and **f** Isolectin B4 at E20, both marking fetal endothelial cells of the fetal blood space of the labyrinth. See Fig. 4 for higher magnification images. *White* (male) and *black* (female). Data shows mean ± SEM, analysed by two-way ANOVAs for sex with zone. *P < 0.05 post-hoc by Student’s *t* test. For E13 *N* = 6–7 samples/sex from 4 litters, E15–*N* = 8–9 samples/sex from 6 litters, E20 *N* = 11–12 samples/sex from 6 litters

**Fig. 4:**
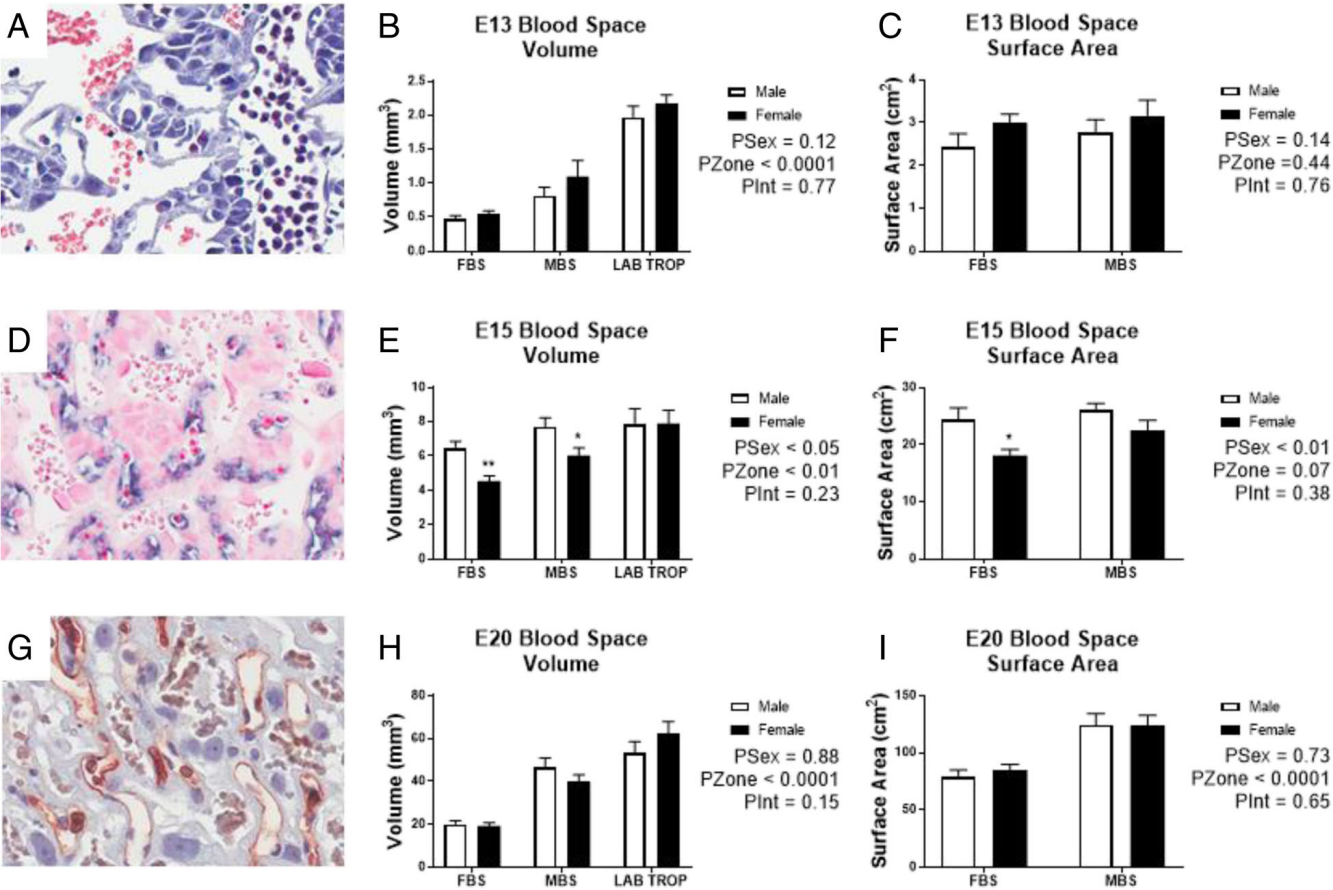
Quantification of labyrinthine blood spaces in males and females in the immature, definitive and late-gestation placenta. **a** Haematoxylin and eosin staining of the E13 labyrinth. **d** In situ hybridisation for *Mest* marking the fetal endothelial cells of the fetal blood spaces in the E15 labyrinth. **g** Isolectin B4 marking the fetal endothelial cells of the E20 labyrinth. *White* (male), *black* (female). Data shows mean ± SEM, analysed by two-way ANOVAs for sex with zone. *P < 0.05, **P < 0.01 from Student’s *t* test. Fetal (*FBS*) and maternal (*MBS*) blood spaces and labyrinth trophoblast (*LAB TROPH*) were estimated for volume for **b** E13, **e** E15 and **h** E20. Similarly, surface areas for FBS and MBS were estimated for **c** E13, **f** E15 and **i** E20. For E13 *N* = 6–7 samples/sex from 4 litters, E15 *N* = 8–9 samples/sex from 6 litters, E20 *N* = 11–12 samples/sex from 6 litters

To further explore the placental compartments in more detail, stereological analysis was carried out at E13, E15 and E20. Placental tissues were stained as outlined in the methods, clearly distinguishing fetal and maternal blood spaces as well as the gross compartments of the labyrinth and junctional zones (see Fig. 3b, d, f and Fig. 4a, d, g for higher magnification). Analysis of total, labyrinth and junctional zone volumes showed each compartment expanded with age (Fig. 3a, c, e). At E13, there was no difference between the sexes in any placental compartment (Fig. 3a). However, the E15 placenta showed marked sex differences; overall, the whole placenta tended to be smaller, but this was not statistically significant (PSex = 0.15). However, the labyrinth volume was lower in females compared to males (*P* < 0.05, Fig. 3c). The LAB:WP ratio was similar between the sexes (data not shown).

**Fig. 5:**
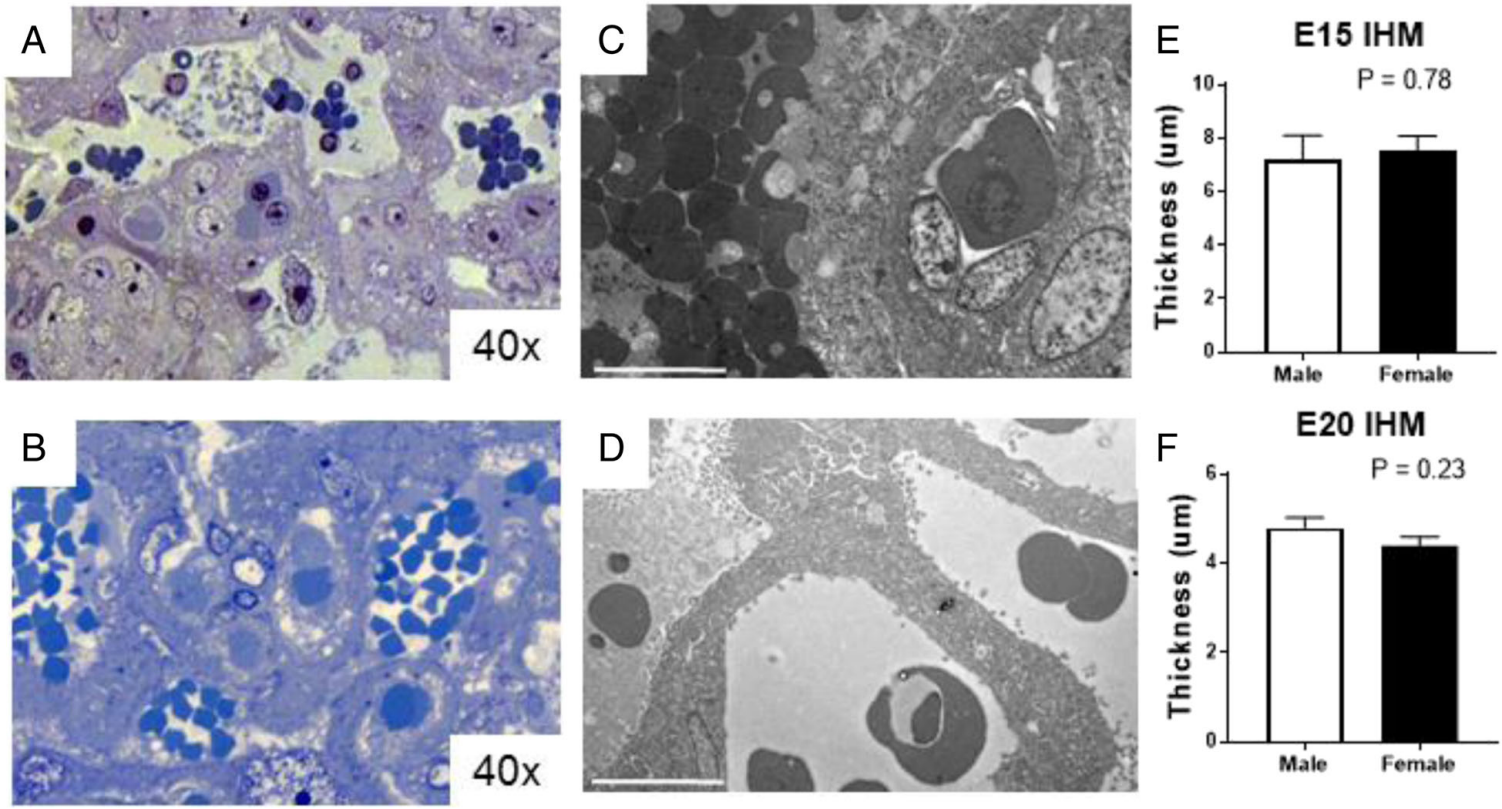
Estimation of interhemal membrane thickness of the male and female labyrinth. Semi-thin sections stained with toluidine blue of the placental labyrinth at **a** E15 and **b** E20. Electron micrographs of the labyrinth at **c** E15 and **d** E20. *White scale bars* in D and E are 10 um. Interhaemal membrane thicknesses at **e** E15 and **f** E20. Data shows mean ± SEM, analysed by Student’s *t* test. *White* (male) and *black* (female). Data averaged 6 individual membranes from 2 blocks. E15 *N* = 5/sex from 3–4 litters. E20 *N* = 4/sex from 4 litters

Given the growth of the labyrinth appeared to be sexually dimorphic, fetal and maternal blood compartments were analysed in more details. Fetal and maternal blood spaces increased with age, but the placenta from females at E15 had a lower volume in both compartments (PSex < 0.05, FBS *P* < 0.01, MBS *P* < 0.05), which was not present earlier (E13) or later (E20) in gestation (Fig. 4b, e, h). The placentas from females also showed reduced blood space surface area (PSex < 0.01), primarily in the fetal compartment at E15 (*P* < 0.05, Fig. 4f). The volume of the labyrinth trophoblast was not different between sexes at any age (Fig. 4b, e, h). Despite marked sex differences in fetal and maternal blood spaces at E15, further structural analysis of the interhaemal membrane showed no differences in thickness at either E15 or E20 between males and females (Fig. 5e, f).

### Labyrinthine expression profiles

**Fig. 6:**
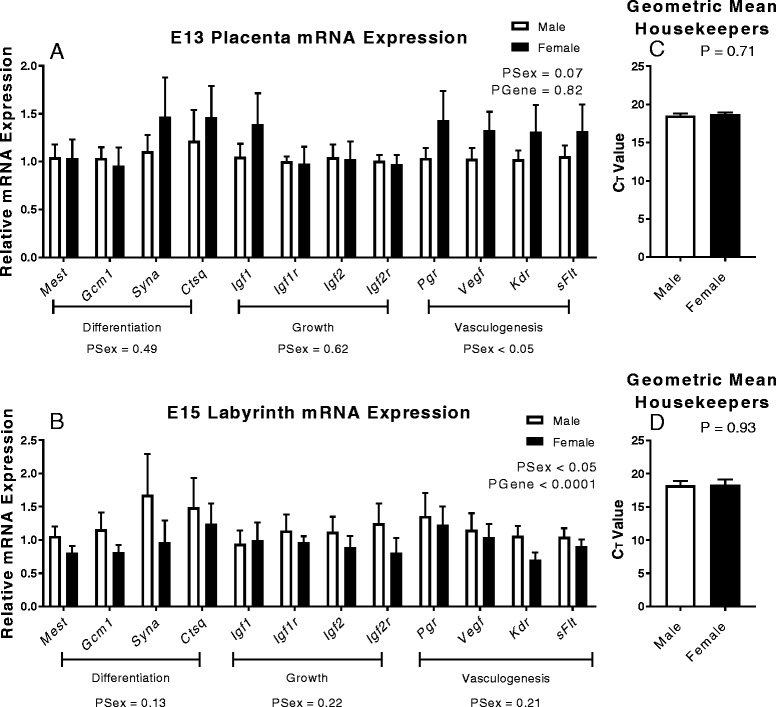
Expression profiles of male and female placentas at E13, and the labyrinth at E15. Expression profiles from whole placenta at **a** E15 and labyrinth samples at **b** E15. Relative expression profiles were calculated relative to the geometric mean of two housekeepers (*Rpl13a*, *18S*) for **c** E13 and **d** E15, with Ct values displayed. *White* (male) and *black* (female). Data shows mean ± SEM analysed by two-way ANOVA for sex with differing gene. Data was standardised to control male at each age. E13–*N* = 8/sex from 4 litters, and E15–*N* = 8/sex from 8 litters

The E15 labyrinth showed clear sexual dimorphism in structure, so next we determined whether there were alterations in expression profiles for genes involved in differentiation, vasculogenesis, growth factors and branching morphogenesis at both E13 and E15 (Fig. 6a, b). Firstly, we determined that expression profiles of many genes increased expression between E13 and E15 (see Additional file 1: Figure S1). Although there were no differences in placental expression of any individual genes between sexes at E13, there was a tendency for an increased in expression of genes in females (PSex = 0.07, Fig. 6a). When analysis was performed on genes grouped into pathways (differentiation, growth and vasculogenesis), a significant increase in expression of genes related to vasculogenesis was found (increases of 20–28%, PSex < 0.05, Fig. 6a). Conversely, E15 female labyrinth samples showed a decrease in expression of the genes investigated (PSex < 0.05, Fig. 6b) with genes regulating differentiation; *Mest*, *Gcm1*, *Syna* and growth; *Igf2* and *Igf2r* reduced by 20–34%. However, this was not statistically significant for individual genes or when analysed for a particular pathway. There was no difference between the Ct values of the geometric mean of the housekeepers at either E13 or E15 (Fig. 6c, d).

## Discussion

This comprehensive ontogeny study has assessed sexual dimorphism in cell number and differentiation of the pre-implantation embryo, and subsequent placental structure throughout gestation in the rat. E15, a time of rapid placental growth in the rat was identified as a critical point during development whereby reduced/slowed differentiation of the placental labyrinth, and its blood compartments in females is likely to be a contributing factor to the lower weight of females by late gestation. This highlights the importance of assessing male and females separately in all developmental studies as differences exist in placental growth, morphology and function even in pregnancies without application of a stressor.

### Pre-implantation development and differentiation

In the blastocyst, no sex differences were found. Cell counts were not different between sexes and were also similar to counts from Wistar rat embryos obtained at a similar time point [41]. Investigation of TE differentiation using culture of in vivo-derived embryos also showed no changes to outgrowth rate, number of trophoblasts, P-TGCs, ploidy or expression of TS and trophoblast differentiation markers. This suggests that males and females have equal developmental potential to differentiate and implant. While this is contrary to the majority of literature which suggests that males are more developed than females, previous studies may be confounded by the use of in vitro fertilisation or superovulation. Indeed, as aforementioned, in vitro-derived embryos show reduced cell counts due to an increase in apoptosis when compared to in vivo-derived embryos [42–44]. Tan et al. [45] recently demonstrated female-specific perturbation of proliferation, and differentiation is caused by the in vitro fertilisation [IVF] procedure. More specifically, they showed IVF in the mouse caused a reduced cell count and increased in apoptosis in female blastocysts during pre-implantation [22]. This was associated with reduced ectoplacental cone formation and elevated mortality by late gestation, resulting in an altered sex ratio at birth (57% male) [22, 45].

However, we do concede that models which do use these in vitro procedures also have shown no differences to cell count and allocation. These include studies using mouse [31], human [23–25], bovine [26–28] and porcine [29, 30] models. Reasons for this could include choice of culture media, such as the addition of serum [27, 28], or the addition [31] or removal [17] of glucose. In humans, as suggested by [24], rather the differences in outcomes may be due to alternate selection criteria for embryo quality prior to transfer, which no longer prioritises the largest blastocysts, which are more likely to be male. Many studies also do not report whether multiple embryos were cultured in the same microdrop, which could affect embryonic growth through the production of autocrine factors.

Artificial reproductive technologies [ART], in addition to IVF, use high, supraphysiological doses of gonadotropins to initiate ovulation. Adverse outcomes are also associated with these procedures, including reduced viability and imprinting disorders [46]. The hormonal contribution to these disorders has been investigated primarily in mice, with gonadotropin administration in vivo being associated with altered genomic imprinting and DNA methylation in offspring [13]. Embryo transfer experiments show that when control derived embryos implant into a hormone-stimulated dam, fetal and placental weights are reduced at E18.5, and do not show any differences in placental DNA methylation profiles [47]. This suggests that gonadotropin exposure can affect both the embryo epigenetic reprogramming and the uterine responses for implantation by different mechanisms, with both affecting long-term fetal and placental development. This highlights the importance of using naturally mated dams without additional hormonal intervention when investigating sex-specific developmental outcomes.

### Placental morphogenesis

To explain the reduced fetal weight in females which is only exhibited in late gestation (E20), the placenta was investigated for sexually dimorphic morphogenic differences at E13, a very early time point of definitive placental development and at E15, a time of rapid placental growth. Analysis of gross placental weight and stereology showed sex-specific structural changes began in the rat at E15. At this age, placental weight was lower in females and this was associated with a smaller relative placental volume and reduced labyrinth vasculature. Further exploration into the labyrinthine blood spaces showed that both the FBS and the MBS were affected. Curiously, while both FBS and MBS showed reduced relative volume in females, only the FBS showed reduced surface area. Overall, this suggests that the female placenta has lower potential capacity for nutrient exchange to facilitate growth at this gestational age.

Investigation of the labyrinthine blood spaces are also of particular importance, as they are also affected in a sexually dimorphic manner in response to maternal perturbations. For example, exposure of mid-late gestational hypoxia shows a decrease in labyrinthine blood spaces in females only, without affecting placental weight in either sex as above [7]. Examination of blood spaces may therefore be a more accurate marker altered placental development. This may suggest other programming models which similarly show placental weight to be unaffected, but do show other changes to gross placental zones, may still have other subtler changes in the vasculature, e.g. glucocorticoid exposure [48]. Sexually dimorphic phenotypes are also likely to be masked by the pooling of sexes in perturbation models, particularly when their affects are divergent.

### Sex differences in placental gene expression

Placentas were investigated for the molecular mechanisms behind this altered labyrinth morphogenesis with selected gene expression assays conducted at E13 and E15. The E13 female placenta showed increased expression of the investigated genes, particularly those associated with vasculogenesis while the E15 female labyrinth decreased gene expression. This was without change to expression of housekeeper genes between males and females. The E13 data is consistent with microarray data on E12.5 mouse placenta, which showed that 183 genes were expressed at a higher level in females whilst only 35 genes were more highly expressed in males [49]. The E15 data coincides with a mouse study at E15.5, which showed that females had greater global DNA methylation profile than males (3.3%) in unstressed conditions [50]. However, the same laboratory has also shown greater upregulation of genes in females than in males at E15.5 [51]. Differences in the expression data at this latter time point are likely due the whole placenta being assayed by Gabory [51], while our study only utilised the labyrinth portion, allowing specific insight into labyrinth development. Collectively, with the structural data, this may suggest that the males may have already undergone the major rounds of proliferation and differentiation by E13, and therefore, by E15 have rather switched to hypertrophic growth which continues to expand the placenta. Future analysis of gene expression profiles of male and placentas at E12–E12.5 would confirm this hypothesis.

### Sexual dimorphism: initiated by the fetal adrenal?

The changes in placental morphogenesis in females only occurred at mid-gestation, which ruled out any contribution from double dosage of the X-chromosome, its inactivation, imprinting and so forth on cellular allocation in the early embryo. A likely candidate contributing to placental changes in mid-gestation is the development and activation of the fetal adrenal gland. Development of the adrenal begins at E10 in the mouse, and its activation contributes to the divergence of ovary and testis developmental at E12.5–13.5 (reviewed by [52]). This in turn upregulates androgen synthesis in XY males promoting further testis development and reciprocal communication with the now masculinised adrenal (reviewed by [53]). Females, however, go down a default morphogenic pathway without hormonal contribution (reviewed by [54]). The growth and development of the male placenta therefore has the potential to react to circulating androgens within the implantation site.

## Conclusions

This study that has identified mid-gestation E15 is an important time point during gestation to analyse basal sex differences, which may not be present in early and late gestation. At this time, the male fetus may have greater metabolic demands than the female fetus, which may lead to the male placenta growing larger and increasing the development and expansion of the fetal and maternal blood spaces in the labyrinth. This in turn, is likely to lead to greater nutrient delivery to the male fetus, and result in a greater fetal weight in late gestation. This greater metabolic demand of the male fetus may also render it more susceptible to subtle changes to the nutrient environment and be more vulnerable to perturbation. As this study showed no basal sex differences in pre-implantation development, this highlights the use of unstressed controls to analyse sexual dimorphism prior to application of a treatment.

## Additional file

Additional file 1: Table S1. Primer pair sequences for genotyping for sex. **Table S2.** Primer pair sequences for in situ hybridisation (ISH) probe synthesis. **Table S3.** SYBR primer sequences qRT-PCR. **Table S4.** TAQman primers for qRT-PCR. **Figure S1.** Temporal gene expression profiles of male and female placentas from E13 to E15. (DOCX 1458 kb)

## Abbreviations

ANOVA: Analysis of variance
ART: Artificial reproductive technologies
DNA: Deoxyribonucleic acid
E: Embryonic day
FBS: Fetal blood space
ICM: Inner cell mass
IHM: Interhemal membrane
IVF: In vitro fertilisation
MBS: Maternal blood space
NHMRC: National health and medical research council
P-TGCs: Parietal trophoblast giant cells
RNA: Ribonucleic acid
RT: Room temperature
TE: Trophectoderm
TS: Trophoblast stem cell
W: Watts
