# Supplementary material for: Sex differences in rat placental development: from pre-implantation to late gestation
Source: Biol Sex Differ. 2017 May 16;8:17. doi: 10.1186/s13293-017-0138-6 (PMC5434533; doi:10.1186/s13293-017-0138-6)
Supplement: Additional file 1: Table S1. — Primer pair sequences for genotyping for sex. Table S2. Primer pair sequences for in situ hybridisation (ISH) probe synthesis. Table S3. SYBR primer sequences qRT-PCR. Table S4. TAQman primers for qRT-PCR. Figure S1. Temporal gene expression profiles of male and female placentas from E13 to E15. (DOCX 1458 kb) [file 13293_2017_138_MOESM1_ESM.docx]

***Additional file 1***

***Supplementary Data***

| ***Gene*** | ***Direction*** | ***Sequence (5’-3’)*** | ***Product (bp)*** |
| --- | --- | --- | --- |
| *Sry* | Forward | TACAGCCTGAGGACATATTA | 317bp |
|  | Reverse | GCACTTTAACCCTTCGATGA |  |
| *B-actin* | Forward | AGCCATGTA CGTAGCCATCC | 220bp |
|  | Reverse | TGTGGTGGTGAAGCTGTAGC |  |

**Table S1. Primer pair sequences for genotyping for sex.** PCR conditions were 95°C for 2 mins, followed by 35 cycles of 95°C for 1 min, 52°C for 1 min, 72°C for 1 min, and extension at 72°C for 5 mins.

| ***Gene*** | ***Direction*** | ***Sequence (5’-3’)*** | ***Product (bp)*** |
| --- | --- | --- | --- |
| *Mest* | Forward (T3) | CTGCTCTGCACTCATGGAAG | 478 |
|  | Reverse (T7) | CCGTCTTTGAGGAGCTTTTG |  |

**Table S2. Primer pair sequences for *In Situ* Hybridisation (ISH) probe synthesis.** Forward primers included the addition of T3 - AATTAACCCTCACTAAAGGG to the 5’ end, and likewise for T7 – TAATACGACTCACTATAGGG.

| ***Gene*** | ***Direction*** | ***Sequence*** |
| --- | --- | --- |
| *Rplp0* | Forward | GAG TGA CAT CGT CTT TAA ACC |
|  | Reverse | AAG CAT TTT GGG TAG TCA TC |
| *Rpl13a* | Forward | GCA CAA GAC CAA AAG AAG |
|  | Reverse | CGC TTT TTC TTG TCA TAG GG |
| *Prl3d1* | Forward | AGA CCT TAT ACA ACA GGA CTC |
|  | Reverse | ATG GCA AAA GAT GAG TGT C |

**Table S3. SYBR primer sequences qRT-PCR.**

| ***Gene of Interest*** | ***ID #*** |
| --- | --- |
| *18S* | 4333760F |
| *Ascl2* | Rn00580387_m1 |
| *Ctsq* | Rn01448613_m1 |
| *Eomes* | Rn01746545_m1 |
| *Flt1* | Rn00570815_m1 |
| *Fgf2r* | Rn01266940_m1 |
| *Gcm1* | Rn00820824_g1 |
| *Hand1* | Rn00572139_m1 |
| *Igf1* | Rn00710306_m1 |
| *Igf1r* | Rn00583837_m1 |
| *Igf2* | Rn01454518_m1 |
| *Igf2r* | Rn01636937_m1 |
| *Kdr* | Rn00564986_m1 |
| *Mest* | Rn01500324_m1 |
| *Pgf* | Rn00585926_m1 |
| *Sry* | Rn04224592_u1 |
| *Syna* | Rn01500024_m1 |
| *Tpbpa* | Rn00597134_g1 |
| *Vegfa* | Rn01511602_m1 |

**Table S4. TAQman primers for qRT-PCR.**

***Supplementary Results***

**Figure S1. Temporal gene expression profiles of male and female placentas from E13 to E15.** All data shows mean ± SEM, standardised to E13 control male. The geometric mean of 2 housekeepers (*Rpl13a, 18S*) were used**.**
